# Tejido tiroideo ectópico mediastínico: Diagnóstico y manejo de una patología infrecuente

**DOI:** 10.23938/ASSN.1128

**Published:** 2025-06-30

**Authors:** Pablo Andrés Ordóñez Lozano

**Affiliations:** Servicio Aragonés de Salud Hospital Universitario Miguel Servet Servicio de Cirugía Torácica Zaragoza España

**Keywords:** Bocio Subesternal, Bocio Nodular, Enfermedades de la Tiroides, Cirugía Torácica Asistida por Video, Goiter Substernal, Goiter Nodular, Thyroid Diseases, Thoracic Surgery Video-Assisted

## Abstract

Los bocios intratorácicos se pueden clasificar en primarios o secundarios. El bocio intratorácico primario, a diferencia del secundario, no tiene conexión con la glándula tiroides cervical. También se han descrito los bocios intratorácicos combinados (presencia sincrónica de bocio intratorácico primario y secundario) que son muy infrecuentes, sugiriéndose la denominación de bocio intratorácico mixto.

Dada la particularidad de estas patologías, se expone el caso de una paciente que presentaba, sincrónicamente, un bocio multinodular y una masa mediastínica de localización paratraqueal derecha correspondiente a tiroides ectópica mediastínica (bocio intratorácico primario). Se describe el proceso diagnóstico diferencial de la masa mediastínica y el manejo realizado mediante cirugía mínimamente invasiva, la cual permitió la exéresis completa de la masa y una rápida recuperación postoperatoria.

## Abstract (en)

Intrathoracic goiters can be classified as primary or secondary. Primary intrathoracic goiters are characterized by the absence of any anatomical connection with the cervical thyroid gland, in contrast to secondary intrathoracic goiters. A rare variant, termed mixed or combined intrathoracic goiter, involves the synchronous presence of both primary and secondary components within the thoracic cavity.

Given the rarity and the diagnostic challenges associated with these conditions, we report the case of a patient who presented with a multinodular goiter and a separate right paratracheal mediastinal mass. The latter was identified as ectopic thyroid tissue, consistent with a primary intrathoracic goiter. We describe the diagnostic approach used to differentiate the mediastinal mass from other thoracic pathologies, as well as the successful surgical management performed via a minimally invasive technique and facilitated a rapid and uneventful postoperative recovery.

## INTRODUCCIÓN

El bocio se define como un agrandamiento de la glándula tiroides al doble de su tamaño normal o a un peso superior a 40 gramos^1^. Sin embargo, no hay una definición de bocio intratorácico (BI) unificada por consenso, existiendo más de diez definiciones en la literatura, similares entre sí^2^. Por lo general, los BI se clasifican en primarios o secundarios. Los BI primarios representan menos del 1% de los BI^1^^,^^2^, y afectan con mayor frecuencia a las mujeres^3^. Los BI secundarios (también denominado bocio cérvico-torácico, bocio subesternal o retroesternal adquirido), son mucho más comunes y su incidencia varía del 2% al 20%, debido a las distintas definiciones utilizadas^1^. También, se han descrito los BI combinados (presencia de BI primario y BI secundario) que son muy infrecuentes^2^. En algunas ocasiones, los nódulos mediastínicos se desarrollan a partir de restos tiroideos en la región tirotímica, mostrando una apariencia similar al de un BI primario^2^, y llegando a presentar en raras ocasiones el crecimiento sincrónico de un nódulo a partir de estos restos tiroideos y el crecimiento de un BI secundario^4^.

Dada la particularidad de estas patologías, que hacen parte de un grupo de situaciones inusuales de presentación de los BI, se expone el caso de una paciente con una masa paratraqueal derecha correspondiente a tiroides ectópica mediastínica, que fue resecada mediante cirugía torácica asistida por video (VATS), y que presentaba sincrónicamente un bocio multinodular, a fin de ilustrar su proceso diagnóstico y manejo.

## CASO CLÍNICO

**Figura 1:**
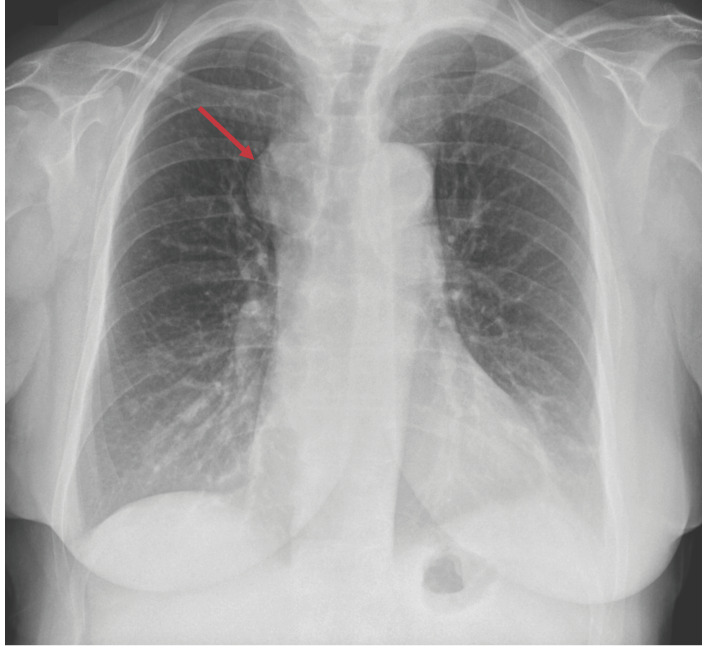
Radiografía de tórax preoperatoria. Se observa ensanchamiento mediastínico relacionado con la masa.

Paciente de 62 años que fue derivada para valoración quirúrgica porque presentaba una masa paratraqueal derecha sugestiva de tiroides ectópica mediastínica, detectada de manera incidental tras visualizarse un ensanchamiento mediastínico en una radiografía (Rx) de tórax (Fig. 1). En el análisis de sangre, el nivel de hormona estimulante de la tiroides (TSH) era normal (0,44 µUI/mL).

**Figura 2:**
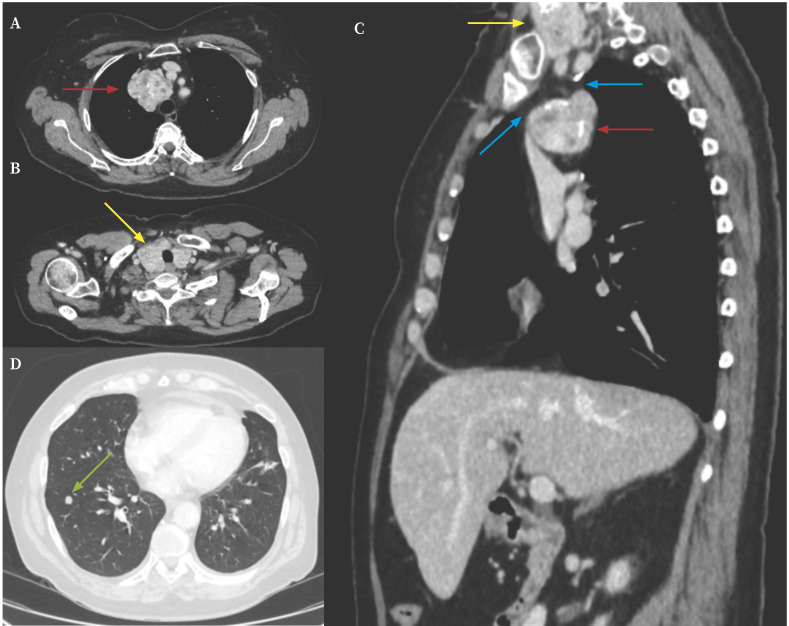
Tomografía computarizada de tórax. **A.** Corte axial a nivel de T4 en ventana de partes blandas. Se observa masa mediastínica (flecha roja) localizada en espacio paratraqueal superior derecho, de contornos polilobulados y con varias calcificaciones que se correlaciona con el ensanchamiento mediastínico de la Rx de tórax. **B.** Corte axial en ventana de partes blandas. Se observa bocio multinodular (flecha amarilla). **C.** Corte sagital. Se observa bocio multinodular (flecha amarilla) y masa mediastínica (flecha roja) sin continuidad entre las lesiones (flecha azul). **D.** Corte axial en ventana de parénquima. Se observa un nódulo pulmonar de 8 mm (flecha verde) en el lóbulo inferior derecho.

En la tomografía computarizada (TC) se observó la presencia de una masa mediastínica localizada en el espacio paratraqueal superior derecho (Fig. 2A) correlacionada con el ensanchamiento mediastínico de la Rx de tórax. De contornos polilobulados y con varias calcificaciones, presentaba unos diámetros axiales de 52 x 46 mm. Producía colapso de la vena braquiocefálica derecha, sin existir signos de infiltración local. Tras la administración de contraste, presentó un realce intenso y heterogéneo, de aspecto multinodular con áreas quísticas y calcificaciones. Su estructura era muy similar a la de la glándula tiroidea/bocio multinodular (Fig. 2B), aunque sin comunicación directa con la misma (Fig. 2C), sugestiva de tiroides ectópica nodular. Además, se informó de la presencia de un nódulo pulmonar solitario de 8 mm de diámetro en el lóbulo inferior derecho, poco denso, y que no tenía características de malignidad (Fig. 2D).

**Figura 3:**
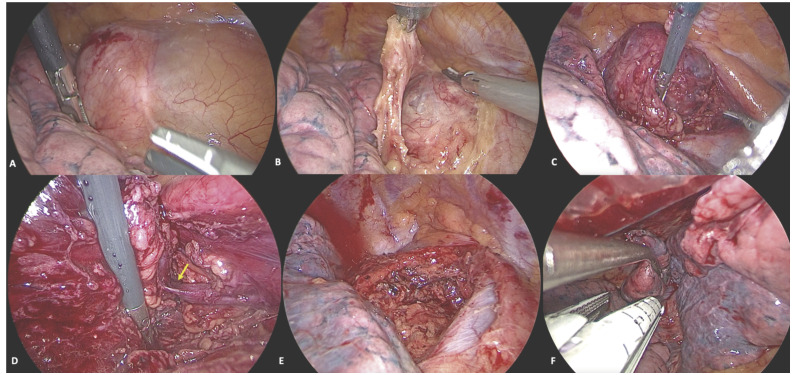
Visión videotoracoscópica. **A.** Al inicio de la cirugía, se observa la masa mediastínica. **B.** Apertura de la pleura mediastínica, paralela al nervio frénico derecho que se respeta. **C.** Disección y sección con bisturí ultrasónico a nivel mediastínico retrocava-paratraqueal. La masa mediastínica presentaba varios vasos sobre su superficie. **D.** Identificación de rama vascular venosa que drena a la vena tiroidea inferior (flecha amarilla), elongada y muy próxima a la masa. **E.** Lecho quirúrgico de resección de la masa mediastínica tras revisión de la hemostasia. **F.** Resección pulmonar sublobar atípica del nódulo pulmonar en lóbulo inferior derecho con endograpadora mecánica.

La exéresis completa de la masa mediastínica se realizó bajo anestesia general e intubación bronquial selectiva, en decúbito semi-supino con el hemitórax elevado a unos 30° desde el plano horizontal. Se llevó a cabo mediante VATS triportal derecha: una incisión de 10 mm a nivel de sexto espacio intercostal con línea axilar media, otra de 5 mm a nivel de cuarto espacio intercostal con línea axilar anterior, y otra de 5 mm a nivel de sexto espacio intercostal con línea medio clavicular, y tras insuflar dióxido de carbono (CO_2_) en la cavidad pleural a 6 mm Hg de presión. La extracción requirió ampliar la incisión localizada a nivel de sexto espacio intercostal con línea medio clavicular y, posteriormente, durante el mismo acto quirúrgico, se realizó una resección sublobar atípica del nódulo pulmonar en el lóbulo inferior derecho (Fig. 3)

Durante su estancia hospitalaria, la paciente cursó con adecuada evolución postoperatoria. Se retiró el drenaje pleural a los tres días de la cirugía, y fue dada de alta hospitalaria.

El informe anatomopatológico describió una masa mediastínica de 54 x 49 x 38 mm diagnosticada de tiroides ectópica con hiperplasia multinodular y calcificaciones, y un nódulo pulmonar de 6 mm en el lóbulo inferior derecho diagnosticado de hamartoma pulmonar.

Tras dos años de seguimiento, la paciente presenta una adecuada evolución clínico-radiológica en relación a la intervención. En la actualidad continúa con controles del bocio multinodular.

## DISCUSIÓN

El tejido tiroideo ectópico (TTE) se define como el tejido tiroideo ubicado en cualquier lugar distinto de su ubicación anatómica normal en el cuello: adyacente a la tráquea, justo por debajo de la laringe^5^. La ubicación más común del TTE es la base de la lengua, pero otras ubicaciones menos frecuentes incluyen la región submandibular o sublingual, laringe, tráquea, esófago, mediastino o incluso el corazón^5^^,^^6^. El TTE mediastínico (como el BI primario), es un subtipo raro de TTE que representa aproximadamente el 1% de los casos de TTE^5^.

El BI primario, a diferencia del secundario, no tiene ninguna conexión fibrosa o parenquimatosa directa con la porción cervical de la glándula tiroides (que es de características normales)^7^. La principal diferencia entre estos dos tipos de bocio es el suministro vascular: mientras el BI primario obtiene su irrigación de los vasos intratorácicos, el secundario conserva su irrigación cervical y muestra continuidad física con cualquier tiroides cervical restante^6^. En este caso, el bocio multinodular y la masa mediastínica de localización paratraqueal no presentaban continuidad entre ellas en la TC. La masa mediastínica, tenía varios vasos sobre su superficie, y destacaba un drenaje venoso a la vena tiroidea inferior.

El BI primario, se origina de las células embrionarias tiroideas ectópicas que han descendido al tórax con el arco aórtico^8^. La mayoría suelen ser asintomáticos con detección incidental en las pruebas radiológicas de tórax, como ocurrió en este caso. En algunos pacientes se ha descrito la compresión de estructuras adyacentes manifestándose con disnea, estridor, tos, sibilancias, síndrome de la vena cava superior, síndrome de Horner, disfagia, o disfonia^9^. La mayoría de los pacientes son eutiroideos^5^^,^^10^; sin embargo, se han descrito casos con hipotiroidismo o hipertiroidismo^5^. En vista del riesgo de compresión de estructuras adyacentes y de la posibilidad, aunque infrecuente^7^, de transformación maligna^5^^,^^9^, la cirugía está indicada tanto en pacientes sintomáticos como asintomáticos.

Las pruebas radiológicas complementarias, como la TC o la resonancia magnética, son útiles en la planificación preoperatoria porque ayudan a definir la relación de la lesión mediastínica con otras estructuras intratorácicas, así como a identificar la irrigación vascular de la lesión^6^ y a distinguir el TTE de otras lesiones que se encuentran en el mediastino^5^. La gammagrafía con yodo radiactivo I-131 puede ser útil ante una alta sospecha clínica de TTE, pero se ha demostrado que el TTE mediastínico tiene una captación limitada que provoca hasta un 10 % de falsos negativos en esta prueba^6^.

Actualmente, y debido a su escasa prevalencia, no existe un consenso sobre el manejo de TTE mediastínico. La mayoría de las publicaciones recomiendan la resección quirúrgica según la edad del paciente, el tamaño de la lesión, los síntomas locales o compresivos, la función tiroidea y las complicaciones de la masa como ulceración o sangrado^5^. Esta resección se ha realizado más frecuentemente mediante abordajes tradicionales como la esternotomía o toracotomía; recientemente se están realizando abordajes mínimamente invasivos, como la VATS^11^ o la cirugía torácica asistida por robot (RATS)^12^. No obstante, el abordaje debe ser seleccionado en función del tamaño, localización, afectación de estructuras locales y experiencia del centro.

El BI secundario se desarrolla por el crecimiento descendente de la glándula cervical (con o sin bocio cervical), cuya vascularización depende de las arterias y venas tiroideas. Este crecimiento y descenso hacia el tórax se ve favorecido por factores anatómicos (estructuras rígidas que delimitan la glándula tiroidea excepto en su límite inferior, presión negativa intratorácica, peso de la glándula y tracción descendente producida por la deglución)^8^. Si hay síntomas, estos están relacionados con la compresión de las vías respiratorias o del esófago. La mayoría de los pacientes presentan una función tiroidea normal, aunque se han descrito casos de hipertiroidismo o hipotiroidismo, y la incidencia de malignidad en estos bocios es similar a la encontrada en los bocios cervicales (entre el 3 y el 21%)^1^. El BI responde escasamente al tratamiento con tiroxina, por lo que la cirugía es el tratamiento de elección y, si es posible, se prefiere la resección total de la glándula mediante abordaje cervical^8^.

También se han descrito los BI combinados, muy infrecuentes, que surgen por extensión subesternal de la tiroides cervical (BI secundario) y el aumento sincrónico de tamaño de la tiroides ectópica mediastínica (BI primario), por lo que se ha sugerido la denominación de BI mixto^2^. En algunas ocasiones, los nódulos mediastínicos se desarrollan a partir de restos tiroideos en la región tirotímica que, o están conectados a la tiroides por una banda fibrosa delgada o no están conectados directamente. Estos nódulos o masas pueden estar asociados con vasos mediastínicos *parásitos* y pueden presentar una apariencia similar al de un BI primario^2^. Rara vez, puede presentarse el crecimiento de un BI secundario y el crecimiento sincrónico de un nódulo a partir de restos tiroideos en la región tirotímica (que no están conectados con la tiroides)^4^.

En el caso de nuestra paciente, hay que destacar que presentaba sincrónicamente un bocio multinodular y una masa mediastínica de localización paratraqueal derecha (poco habitual) correspondiente a tiroides ectópica mediastínica (BI primario). Además, el manejo realizado mediante VATS permitió la exéresis completa de la masa y la rápida recuperación postoperatoria propia de una técnica quirúrgica mínimamente invasiva. La correcta interpretación de las relaciones anatómicas (principalmente vasculares) de la lesión en las pruebas radiológicas es indispensable para seleccionar la mejor vía de abordaje y planificación quirúrgica.

## Data Availability

Se encuentran disponibles bajo petición al autor de correspondencia.
